# The effects of emotions on the disposition to normative and non-normative political action in the context of the Chilean post-social outburst

**DOI:** 10.3389/fpsyg.2023.1154501

**Published:** 2023-06-21

**Authors:** Fuad Hatibovic, Juan Sandoval, Ximena Faúndez, Jose-Manuel Gaete, Magdalena Bobowik, Paola Ilabaca

**Affiliations:** ^1^Escuela de Psicología, Facultad de Ciencias Sociales, Universidad de Valparaíso, Valparaíso, Chile; ^2^Escuela de Sociología, Facultad de Ciencias Sociales, Universidad de Valparaíso, Valparaíso, Chile; ^3^Ramón y Cajal Research Fellow at Universidad del País Vasco/Euskal Herriko Unibertsitatea, País Vasco, Spain; ^4^School of Psychology, School of Juridical and Social Sciences, Miraflores Campus, Universidad Viña del Mar, Viña del Mar, Chile

**Keywords:** positive emotions, negative emotions, normative political action, non-normative political action, Chilean social outburst

## Abstract

This article analyzes the role of Chileans’ emotions as predictors of normative and non-normative political action in the context of the post-social outbreak and the constituent process. We carried out three descriptive studies: first, a study conducted 1 year after the social outburst (*n* = 607), a second one carried out before the constitutional referendum (*n* = 320), and a third study conducted after the constitutional referendum (*n* = 210). The results indicated that participants present a higher disposition to normative over non-normative political action, although both lose strength as the studies temporally move away from the social outburst. Also, our research established that emotions directed towards different events related to the Chilean political process play a conspicuous role in predicting the disposition to mobilize in a normative and non-normative way.

## 1. Introduction

In Chile, October 18, 2019, marked the beginning of the so-called “social outburst” characterized by massive mobilizations and a broad malaise on the part of multiple players who demanded substantial changes to the country’s economic and social model (Araujo, 2019). Although the immediate cause was the public transportation fare hike, the popular rallies soon exposed its deeper causes. That is, the existence of several social problems affecting the vast majority of the population, namely: the high cost of living, deficit health system, low pensions, generalized rejection of the political class, and institutional discredit accumulated during the last years, including the 1980 Political Constitution imposed by Augusto Pinochet and reformed in post-dictatorship (Folchi, 2019).

The demonstrations spread throughout the national territory, originating a response with high levels of violence from State agents, specifically from the Armed Forces and Law Enforcement (Amnesty International, 2019; Organización de las Naciones Unidas, 2019; Inter-American Commission on Human Rights, 2020). This scenario blew after the decree of a State of Constitutional Emergency Exception, which limited, for example, the right to peaceful assembly.

In a framework of massive mobilizations and a high level of police repression, on November 15, 2019, representatives of the ruling party and the opposition signed the “Agreement for Peace and the New Constitution,” which accelerated the process of creating a new constitution. Thus, on April 26, 2020, an initial referendum was held so that the population could vote “I approve” or “I reject” to change the Political Constitution of 1980, inherited from the civil-military dictatorship, winning “I approve.” Subsequently, the Constitutional Convention (which functioned between July 4, 2021, and July 4, 2022) was elected. This entity was in charge of drafting a proposal for the Political Constitution of the Republic. On September 4, 2022, Chilean citizens voted in a last referendum to determine whether they agreed or disagreed with the proposed new constitution. Approximately 13 million Chileans voted in this referendum; the rejection side obtained 61.8% of votes over 38.13% approval. This fact constituted a heavy defeat for Chile’s progressive forces.

### 1.1. Social outburst in Chile

In the 2019 demonstrations, protesters demanded diverse social claims articulated in the collective action cycle of the last decade. Since the mid-2000s, Chile has experienced the rise of new forms of political action and organization, which achieved public visibility from the student mobilizations of 2011 (García and Aguirre, 2014; Sandoval and Carvallo, 2019). From that date, it is possible to recognize a cycle of re-politicization of everyday life (Zarzuri, 2021), in which students, inhabitants of extreme regions, activists of environmental movements, and feminist collectives are its protagonists (Salinas, 2016). In this cycle, conventional and unconventional political practices were incubated and deployed with maximum intensity and amplitude during the social outburst (Cortes, 2022).

Historically, this cycle of mobilizations occurs in a context of questioning the political and economic system consolidated in the Chilean post-dictatorship (Pereda-Pérez and Howard, 2015). The manifestations of the social outburst denounced the high levels of economic inequality (PNUD, 2018), added to territorial, age, and gender inequalities, but also exposed the crisis of representation, trust, and probity of the political system (Morales, 2020). Therefore, we should understand the Chilean revolt as the expression of a sustained accumulation of malaise, distrust in institutions, a crisis of representativeness, and an increased perception of corruption (Moyano-Díaz et al., 2021).

Therefore, the social outburst represents the connection of ordinary people who recognized themselves in moral indignation in the face of inequality (Canales, 2022). It was not an ideological discourse that criticized the inequality of the system but the transition from diffuse discomfort to concrete indignation (Paredes and Valenzuela Fuentes, 2020) that changed the way of experiencing the malaise that had been accumulating for decades. As shown by an increasingly relevant line of research in the social sciences (Poma and Gravante, 2017a), emotions played a fundamental role in the origin and form of the October social revolt.

In the literature on social outburst, it is possible to find several works focused on emotions. For example, some authors described the outbreak as the result of the accumulated discomfort that turned into rage and indignation from the gap between subjectivity and social structure (Mayol, 2019; Martuccelli, 2021). Other authors have analyzed how the contexts of mobilization trigger a battery of emotions, describing cultural consequences in the rules of feeling that favored the demonstrations of 2019 (Paredes and Valenzuela Fuentes, 2020). Another author claimed that the social revolt generated an experience of emotional ambivalence between joy for the creative and hopeful dimension of the protests and rage for the repressive response of the state (Sandoval, 2021). Following this line of argument, other authors described that emotions -particularly unpleasant ones-play a relevant role in the willingness to attend protests, while pleasant emotions experienced during demonstrations are related to the commitment to continue participating in these types of actions (Asún et al., 2021).

This article aims to contribute to studies on emotions, empirically addressing the place of positive and negative feelings in the willingness to participate in political actions in the context of the post-social outburst.

### 1.2. The forms of political action

One of the ways of classifying political action is the distinction between conventional and unconventional political action. For Barnes et al. (1979), who propose this distinction, conventional political participation is related to electoral processes, while unconventional participation is related to acts such as signing petition letters, legal demonstrations, property damage, or violent actions. According to Sabucedo and Arce (1991), both forms of political participation are differentiated by the type of demand they make on the political system: through the constituted power in conventional action, and through confrontation with the legality in non-conventional action.

Some researchers have problematized this taxonomy beyond the canonical character of the conventional-unconventional distinction. Authors have questioned its dichotomous nature (Sabucedo and Arce, 1991; Delfino and Zubieta, 2010; Sorribas and Brussino, 2013a), its ability to discriminate new forms of action (Morales, 2005), and its effectiveness in the face of new theoretical nomenclatures (Sandoval et al., 2012). Respecting conventional participation, some authors discussed the differentiated nature of voting behavior concerning the rest of institutionalized political actions (Delfino and Zubieta, 2010); and others, the unidimensional nature of the measurement scales (Brussino et al., 2013). Regarding unconventional actions, mainly the debate is about the heterogeneous character of the activities included in this category (Sabucedo and Arce, 1991), particularly violent actions (Delfino and Zubieta, 2014).

Notwithstanding the above, empirical results at the international level continue to confirm the existence of two forms of political action (DiGrazia, 2014), one oriented to the representation system (Sorribas and Brussino, 2013b) and the other non-institutional (Quaranta, 2012). New studies address these forms of action through different nomenclatures, such as the dichotomy between normative and non-normative action (Tausch et al., 2011) or between institutional participation and direct participation (Delfino et al., 2013).

This paper assumes a two-dimensional concept of political action, normative and non-normative (Tausch et al., 2011; Sandoval et al., 2018). Normative forms of political action include conventional and non-conventional activities of legal nature associated with different dimensions of institutionality (Tausch et al., 2011). As the literature argues (Muller, 1982; Sabucedo and Arce, 1991; Rucht, 1992), this dimension confirms the integrity of a type of political action organized around its systemic legality. Non-normative political action, on the other hand, groups together forms of direct action defined in opposition to the normative order and that occasionally may exceed legality (Tausch et al., 2011). According to Melucci’s (1996) proposal, this dimension confirms a class of political action defined by an unmediated confrontation with the system. Also, it set up the typical repertoire of belligerent actions or social protests (Sorribas and Brussino, 2013a) that predominated during the social outburst of 2019.

### 1.3. Emotions and political action

Since the late 20th century, a systematic line of research on emotions and their influence on protest actions began to take shape. In recent decades (Poma and Gravante, 2017a), empirical research has been developing on the role of displeasing or negative emotions (anger, fear, indignation) and pleasant or positive emotions (hope, pride, joy) (Reisenzein, 1994) in the emergence and maintenance of protest cycles (Reed, 2004; Bosco, 2006; Poma and Gravante, 2018).

As Jasper (2014) indicates, we rarely experience emotions in isolation, much less in a context of social mobilization, where emotions such as fear, anger, and joy may be present at the same time. To address the above, the author proposes working with pairs of emotions (positive–negative) or “moral batteries,” which would give us an account of the tendency of subjects to mobilize towards a desired goal and to move away from what is threatening or unpleasant. This fact allows us to observe, for example, how anger can facilitate the transformation from shame to pride, as in the case of the gay and lesbian movement (Whittier, 2012) or how hope and indignation were used in the discourses of the anarchist resistance in Spain under Franco’s dictatorship (Romanos, 2014).

Studies on emotions and collective action have also addressed the relation that some feelings establish with protest actions. In the case of positive emotions, they have been studied in the context of social mobilizations, showing the facilitating character that hope, for example, would have in the maintenance and development of protest actions (Williamson, 2011; Van Troost et al., 2013). In this regard, Reed (2004) describes how hope functions as an accelerator, representing a way to channel negative emotions such as anger, indignation, and even fear, facilitating the legitimization of individual and collective actions involved in protests. However, when hope and collective action are mediated by collective motivation, it has been found that there is no significant indirect effect (van Zomeren et al., 2019). Some authors have described positive feelings toward the Black Lives Matter Movement as mediating between collective efficacy and collective action intentions, providing evidence for the role of these emotions in collective action intentions (Lizarazo Pereira et al., 2022).

On the other hand, antagonistic negative emotions, such as anger or rage in situations perceived as unfair or undesirable, have a relevant impact on the willingness to mobilize (Flam, 2014; Jasper, 2014; Poma and Gravante, 2016), playing an important role in the transformation of shame into pride, and in the defiant disposition at the fore of a threatening enemy or authority (Smith and Lazarus, 1990; Klandermans et al., 2008). Conversely, when anger or rage is not directed towards a clear and specific goal, it tends to manifest as anxiety, not in a way beneficial to the movement and the individuals participating in it (Van Ness and Summers-Effler, 2018). Also, increased group anger has been found to be a predictor of future collective action intentions (Tausch and Becker, 2013; Radke et al., 2022). On the other hand, it has been found that anger can be more relevant to collective action when activists are members of a disadvantaged group (Landmann and Rohmann, 2020).

Finally, negative emotions of resignation, such as fear, also influence forms of political action (Williamson, 2011; Reed, 2014; Rigby and Sørensen, 2017; Ransan-Cooper et al., 2018). In this regard, some authors described that fear could act by inhibiting action (Poma and Gravante, 2017b) or motivating it (Reed, 2014). Fear could facilitate the union and formation of groups willing to carry out resistance actions. It is the case of the anti-coal seam gas (anti-CSG) movement in Australia, where fear and anger at the possibility of installing this type of industry played a crucial role in the origin of activist groups. The merge of these emotions with other positive feelings makes it possible to sustain protest actions over time, such as the love for the places defended and the enjoyment of the social connection that emerges during mobilizations (Ransan-Cooper et al., 2018). Moreover, it has been found that the motivation to instill fear of the outgroup was related to violent action. (Hasan-Aslih et al., 2019).

As seen above, the available literature on the role of emotions in protest actions is significant, highlighting the study of emotions in pairs, batteries, or chains of positive and negative emotions (Jasper, 2011). From the theoretical review, in this paper, we ask ourselves about the relation between positive and negative emotions (antagonistic or resignation) and forms of political action -normative and non-normative- that emerged after the social outburst of October 2019. We conducted 3 descriptive correlational studies to achieve this purpose, developed at 3 different moments relevant to the Chilean political process: (a) the commemoration of the first year after the social outburst (study 1), (b) 2 months before the Chilean constitutional plebiscite (study 2), and (c) a month and a half after the triumph of the rejection option in the plebiscite (study 3).

### 1.4. Objectives and hypotheses

Objectives:

1. To determine the predictive value of negative antagonistic and negative emotions of resignation and positive emotions on normative and non-normative political action.
2. To investigate the incidence of antagonistic negative emotions and resignation and positive emotions before relevant milestones as mediating variables in the relation between emotions towards the political system and protests – predictive variables – with normative political action and non-normative political action – criterion variables.

Based on the theoretical review, the following hypotheses are proposed:

1. Antagonistic negative emotions during the social outburst and toward police repression will predict a higher disposition toward normative and non-normative political action.
2. Positive emotions during the social outburst and toward protests will predict a higher disposition toward normative and non-normative political action.
3. Antagonistic negative emotions toward police repression and corruption and negative emotions of resignation toward police repression mediate the relation between hope toward protests and normative political action and non-normative political action.
4. Negative antagonistic and resigned emotions and positive feelings towards the triumph of the rejection option in the referendum and towards the protests will mediate the relation between antagonistic negative emotions towards the political system and normative political action and non-normative political action.

## 2. Study 1

### 2.1. Method

#### 2.1.1. Participants and procedure

The study participants were 607 Chileans with an average age of 28.02 years (SD = 12.21), of whom 72.5% were female, 24.4% were male and 3.2% identified with another gender. The participants were from different regions of Chile (34% from the Valparaíso region; 25.6% from the Metropolitan region; 11.6% from the Libertador General Bernardo O’Higgins region and 11.7% from the Bio Bio region). Regarding their political position, 34.7% considered themselves left-wing, while 42.4% said they had no political position and 9% considered themselves right-wing. Regarding religious orientation, 32.9% were Catholic, 50.5% did not belong to any religion and 16.5% were of other religions.

Participants were invited to fill in an online questionnaire via the SurveyMonkey@platform. Participants were recruited by snowballing sampling procedure. That is, the invitation to fill in the survey was distribute through e-mail and using social networks such as Facebook, Instagram, and Twitter. The data collection started on October 18, 2020, 1 year after the social outburst in Chile, and was extended for 4 weeks until November 15 of the same year.

#### 2.1.2. Measures

***Antagonistic negative emotions during the social outburst***

Participants were asked to indicate on a scale ranging from 1 (*not at all*) to 7 (*a lot*) to what extent they experimented “anger,” “hatred,” “annoyance” and “rage” during the social outburst (*α* = 0.85).

***Negative emotions of resignation during the social outburst***

We asked participants to report on a scale ranging from 1 (*not at all*) to 7 (*a lot*) to what extent they felt “fear,” “dread,” and “nervousness” during the social outburst (*α* = 0.86).

***Positive emotions during the outburst***

We asked participants to report on a scale ranging from 1 (*not at all*) to 7 (*a lot*) to what extent they felt hope, joy, pride and interest (*α* = 0.82).

***Negative emotions antagonistic towards protests***

Participants were asked to indicate on a scale ranging from 1 (*not at all*) to 7 (*a lot*) to what extent they experimented “anger,” “hatred,” “annoyance” and “rage” towards protests (*α* = 0.90).

***Negative emotions of resignation towards protests***

We asked participants to report on a scale ranging from 1 (*not at all*) to 7 (*a lot*) to what extent they felt “fear,” “dread,” and “nervousness” towards protests (*α* = 0.82).

***Positive emotions towards protests***

We asked participants to report on a scale ranging from 1 (*not at all*) to 7 (*a lot*) to what extent they felt hope, joy, pride, interest, sympathy, empathy and respect (*α* = 0.94).

***Negative emotions antagonistic towards police repression***

Participants were asked to indicate on a scale ranging from 1 (*not at all*) to 7 (*a lot*) to what extent they experimented “anger,” “hatred,” “annoyance” and “rage” to police repression (*α* = 0.93).

***Negative emotions of resignation towards police repression***

We asked participants to report on a scale ranging from 1 (*not at all*) to 7 (*a lot*) to what extent they felt “fear,” “dread,” and “nervousness” towards police repression (*α* = 0.92).

***Positive emotions towards police repression***

We asked participants to report on a scale ranging from 1 (*not at all*) to 7 (*a lot*) to what extent they felt joy, pride and sympathy (*α* = 0.88).

***Normative political action***

We captured intentions to participate in non-normative action by asking participants about their general willingness to participate in political activities in Chile. Specifically, they were asked to indicate on a scale from 1 (*not willing at all*) to 7 (*extremely willing*) to what extent they would be willing to “Sign a petition,” “Participate in legal/sanctioned demonstrations (marches),” “Give opinions about politics on social networks (Twitter, Facebook, etc.),” and “Vote in municipal, parliamentary or presidential elections.” The scale was reliable (*α* = 0.73).

***Non-normative political action***

Non-normative collective action intentions were measured with other four items. Participants responded on a scale from 1 (*not willing at all*) to 7 (*extremely willing*) to what extent they would be willing to “Support boycotts,” “Participate in illegal strikes (work stoppages),” “Occupy buildings or factories (seizure),” “Participate in violent actions such as throwing stones, burning or breaking urban furniture, barricades, etc..” The scale showed good internal consistency (*α* = 0.84).

#### 2.1.3. Analytical strategy

**Figure 1:**
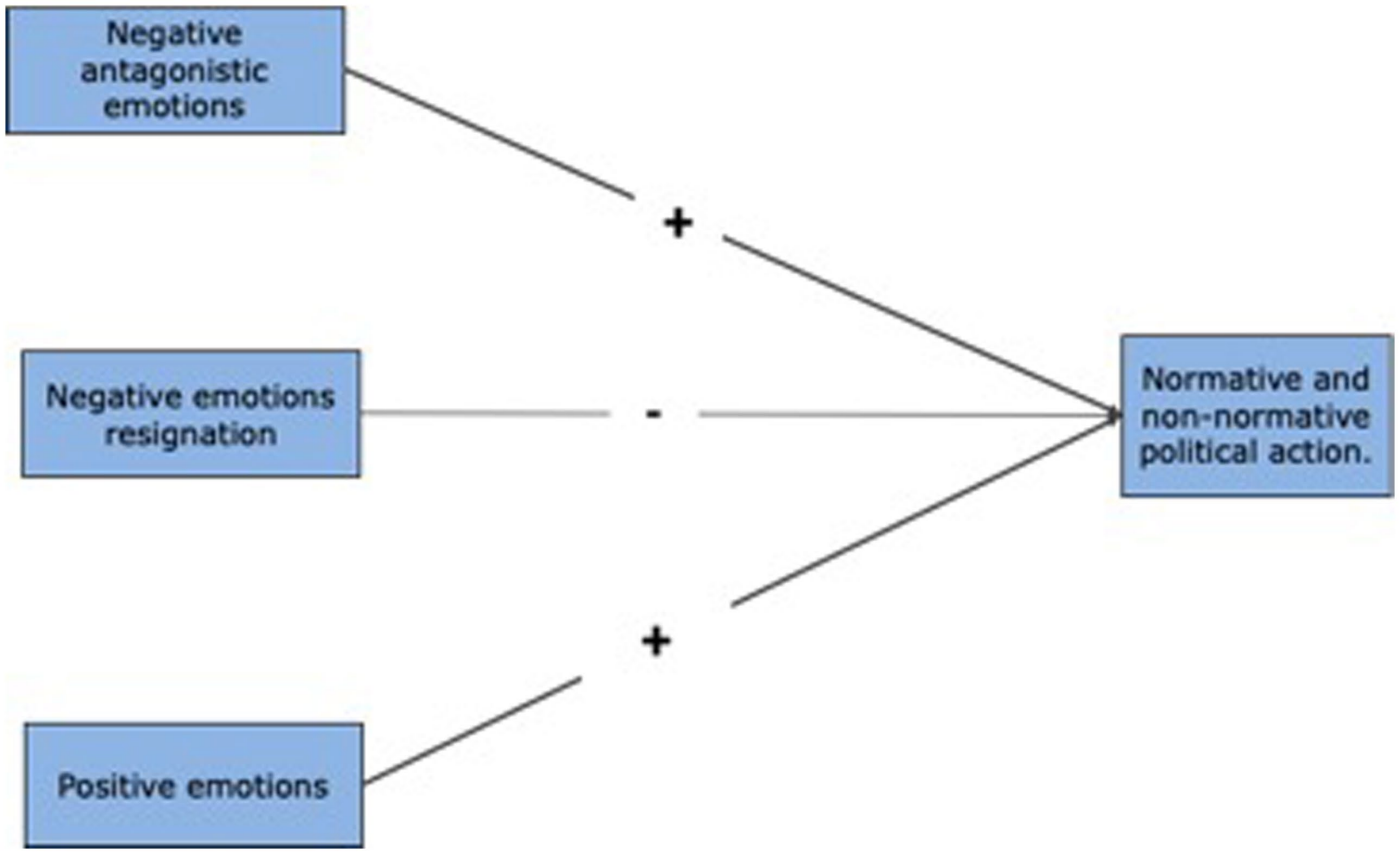
Theoretical model of regression analysis.

We used Pearson’s coefficient to calculate the correlation. We used multiple regression analysis to predict the relation between variables. We set the disposition to political action (normative and non-normative) as the criterion variable and variables measuring emotions as predictor variables in different regression models. We used SPSS software, version 24. The regression model is based on a predictive relationship between the predictor variables (emotions) and the criterion variables (normative and non-normative political action), as posited in the reviewed literature and illustrated in Figure 1.

### 2.2. Results

#### 2.2.1. Correlation analysis

We found a significant correlation between most variables analyzed. As Table 1 shows, normative political action was positively associated with antagonistic negative emotions during the outburst *r*(555) = 0.31, *p* < 0.01 and positive emotions toward protests *r*(558) = 0.61, *p* < 0.01; and was negatively associated with antagonistic negative emotions toward protests *r*(558) = −0.25, *p* < 0.01 and positive emotions toward police repression *r*(555) = −0.20, *p* < 0.01.

**Table 1:** Means, standard deviations, and correlations.

Variable	M	SD	1	2	3	4	5	6	7	8	9	10
1. Normative political action	5.76	1.32	1									
2. Non-normative political action	3.25	1.79	0.55^**^	1								
3. Negative antagonistic emotions during the outburst	4.71	1.64	0.31^**^	0.38^**^	1							
4. Negative emotions resignation during the outburst	4.68	1.61	0.11^*^	0.04	0.35^**^	1						
5. Positive emotions during the outburst	4.94	1.57	0.54^**^	0.46^**^	0.12^**^	−0.05	1					
6. Negative emotions antagonistic to protests	2.53	1.72	−0.25^**^	−0.18^**^	0.24^**^	0.16^**^	−0.30^**^	1				
7. Negative emotions of resignation towards protests	3.48	1.66	−0.07	−0.12^**^	0.17^**^	0.60^**^	−0.16^**^	0.47^**^	1			
8. Positive emotions towards protests	5.27	1.71	0.61^**^	0.53^**^	0.20^**^	0.01	0.79^**^	−0.46^**^	−0.21^**^	1		
9. Negative emotions antagonistic to police repression	5.61	1.82	0.62^**^	0.56^**^	0.40^**^	0.13^**^	0.56^**^	−0.25^**^	−0.04	−0.21^**^	1	
10. Negative emotions resignation towards police repression	5.12	1.88	0.45^**^	0.29^**^	0.19^**^	0.48^**^	0.40^**^	−0.18^**^	0.26^**^	0.49^**^	0.63^**^	1
11. Positive emotions towards police repression	1.46	1.16	−20^**^	−0.18^**^	0.03	−0.04	−0.20^**^	0.33^**^	0.11^**^	−0.26^**^	−0.40^**^	−0.33^**^

M and SD are used to represent mean and standard deviation, respectively. ^*^ indicates *p* < 0.05. ^**^ indicates *p* < 0.01.

On the other hand, we found a significant association between non-normative political action with antagonistic negative emotions toward police repression *r*(548) = 0.56, *p* < 0.01 and with positive emotions toward protests *r*(552) = 0.53, *p* < 0.01. And non-normative political action was negatively associated with antagonistic negative emotions toward protests *r*(552) = −0.18, *p* < 0.01 and with positive emotions toward police repression *r*(549) = −0.18, *p* < 0.01.

Based on these results, we can establish that the variables are independent, which allows us to perform regression analyses to investigate the role of emotions as explanatory variables of the disposition to political action (normative and non-normative).

#### 2.2.2. Regression analysis

These regression analyses allow us to determine the effects of negative emotions (antagonistic and resignation) and positive emotions during the outburst towards protests and police repression on normative and non-normative political action. To accomplish this, we presented four regression models below (see Table 2).

**Table 2:** Linear regression analysis.

	Non-normative political action (Model 1)			Non-normative political action non-normative political action (Model 2)			Normative political action (Model 3)			Normative political action (Model 4)		
	B	SE	*β*	B	SE	*β*	B	SE	*β*	B	SE	*β*
Negative antagonistic emotions during the outburst	0.39	0.04	0.13^***^	0.27	0.05	0.24^***^	0.19	0.03	0.23^***^	0.11	0.03	0.13^**^
Negative emotions resignation during the outburst	−0.07	0.04	−0.06	0.01	0.06	0.01	0.04	0.03	0.05	0.01	0.04	0.01
Positive emotions during the outburst	0.48	0.04	0.42^***^	0.16	0.07	0.14^*^	0.44	0.03	0.52^***^	0.16	0.05	0.19^**^
Negative emotions antagonistic to protests				−0.01	0.05	−0.01				−0.05	0.04	−0.06
Negative emotions of resignation towards protests				−0.09	0.05	−0.08				−0.01	0.04	−0.02
Positive emotions towards protests				0.20	0.08	0.19^**^				0.13	0.05	0.17^*^
Negative emotions antagonistic to police repression				0.29	0.06	0.29^***^				0.23	0.04	0.32^***^
Negative emotions resignation towards police repression				−0.07	0.05	−0.08				0.05	0.04	0.06
Positive emotions towards police repression				0.01	0.06	0.00				0.08	0.04	0.07
Total F		86.59^***^			38.98^***^			102.82^***^			53.75^***^	
*R*^2^ adjusted	0.32				0.39			0.36			0.47	

^***^p < 0.01, ^**^p < 0.01, and ^*^p < 0.05.

##### 2.2.2.1. Non-normative political action

In model 1, the results show that antagonistic negative emotions during the outburst *β* = 0.13, *t*(537) = 18.02, *p* < 0.001 have a significant effect on non-normative political action, as do positive emotions during the outburst *β* = 0.42, *t*(537) = −11.79, *p* < 0.001. The *R*^2^ value was 0.32. Therefore, the model would explain 32% of the total variance. On the other hand, Model 2 also shows that antagonistic negative emotions during the outburst *β* = 0.24, *t*(516) = 5.43, *p* < 0.001 and positive emotions during the outburst *β* = 0.14, *t*(516) = 2.37, *p* < 0.05, have a significant effect on the criterion variable. Further, when we introduced new predictor variables, positive emotions toward protests *β* = 0.19, *t*(516) = 2.65, *p* < 0.01 and antagonistic negative emotions toward police repression *β* = 0.29, *t*(516) = 4.71, *p* < 0.001 have a significant effect on non-normative political action, increasing the *R*^2^ value to 0.39. This second model would explain 39% of the total variance.

##### 2.2.2.2. Normative political action

In model 3, the results show that antagonistic negative emotions during the outburst *β* = 0.23, *t*(543) = 6.15, *p* < 0.001 has a significant effect on normative political action, as does positive emotions during the outburst *β* = 0.52, *t*(543) = 15.13, *p* < 0.001. The *R*^2^ value was 0.36, indicating that the model would explain 36% of the total variance. On the other hand, in Model 4, it is also shown that antagonistic negative emotions during the burst *β* = 0.13, *t*(522) = 3.13, *p* < 0.001 and positive emotions during the burst *β* = 0.19, *t*(522) = 3.47, *p* < 0.01, have a significant effect on the criterion variable. On the other hand, when we introduced new predictor variables, positive emotions toward protests *β* = 0.17, *t*(522) = 2.57, *p* < 0.05, and antagonistic negative emotions toward police repression *β* = 0.32, *t*(522) = 5.47, *p* < 0.001 have a significant effect on normative political action, increasing the *R*^2^ value to 0.47. This fourth model would explain 47% of the total variance.

## 3. Study 2

### 3.1. Method

#### 3.1.1. Participants and procedure

The study participants were 320 Chileans with an average age of 35.51 years (SD = 11.08), of whom 45.6% were female, 52.6% were male and 1.0% identified with another gender. The participants were from different regions of Chile (36.8% from the Valparaíso region; 31.3% from the Metropolitan region). Participants were invited to fill in an online questionnaire via the SurveyMonkey@ platform. Participants were recruited by snowballing sampling procedure. That is, the invitation to fill in the survey was distribute through e-mail and using social networks such as Facebook, Instagram, and Twitter. The data collection started on July, 2022, before the Chilean constitutional plebiscite^1^, and was extended until September 4 of the same year.

#### 3.1.2. Measures

***Negative emotions antagonistic towards police repression***

Participants were asked to indicate on a scale ranging from 1 (*not at all*) to 7 (*a lot*) to what extent they experimented “anger,” “indignation,” “annoyance” and “rage” to police repression (*α* = 0.98)

***Negative emotions of resignation towards police repression***

We asked participants to report on a scale ranging from 1 (*not at all*) to 7 (*a lot*) to what extent they felt “fear,” “dread,” and “nervousness” towards police repression (*α* = 0.96).

***Negative emotions antagonistic towards corruption***

Participants were asked to indicate on a scale ranging from 1 (*not at all*) to 7 (*a lot*) to what extent they experimented “anger,” “hatred,” “annoyance” and “indignation” towards corruption. These four items were used as an overall indicator of negative emotions antagonistic towards corruption (*α* = 0.91).

***Hope towards protests***

Participants were asked to indicate on a scale ranging from 1 (*not at all*) to 7 (*a lot*) to what extent they experimented hope towards protests.

***Normative political action***

We captured intentions to participate in non-normative action by asking participants about their general willingness to participate in political activities in Chile. Specifically, they were asked to indicate on a scale from 1 (*not willing at all*) to 7 (*extremely willing*) to what extent they would be willing to “Sign a petition,” “Participate in legal/sanctioned demonstrations (marches),” “Give opinions about politics on social networks (Twitter, Facebook, etc.),” and “Vote in municipal, parliamentary or presidential elections” (*α* = 0.61).

***Non-normative political action***

Non-normative collective action intentions were measured with other four items. Participants responded on a scale from 1 (*not willing at all*) to 7 (*extremely willing*) to what extent they would be willing to “Support boycotts,” “Participate in illegal strikes (work stoppages),” “Occupy buildings or factories (seizure),” “Participate in violent actions such as throwing stones, burning or breaking urban furniture, barricades, etc..” The scale showed good internal consistency (*α* = 0.81).

#### 3.1.3. Analytical strategy

We used Pearson’s coefficient for correlation analyses. We also used the Process v3.5 macro of SPSS version 24, with the multiple mediation model that simultaneously estimates multiple indirect effects with their standard errors and confidence intervals derived from the Bootstrap distribution (Preacher and Hayes, 2004).

### 3.2. Results

Regarding the association between the variables analyzed, we found a significant correlation between all variables. As Table 3 shows, normative political action was positively associated with antagonistic negative emotions toward police repression *r*(317) = 0.52, *p* < 0.01, negative feelings of resignation toward police repression *r*(317) = 0.37, *p* < 0.05, and hope toward protests *r*(317) = 0.52, *p* < 0.01.

**Table 3:** Means, standard deviations, and correlations.

Variable	M	SD	1	2	3	4	5	6
1. Normative political action	5.48	1.56	1					
2. Non-normative political action	2.76	1.88	0.43^**^	1				
3. Antagonistic negative emotions police repression	5.04	2.35	0.52^**^	0.53^**^	1			
4. Negative emotions resignation police repression	4.41	2.30	0.37^*^	0.33^**^	0.77^**^	1		
5. Negative emotions antagonistic corruption	6.23	1.30	0.24^**^	0.17^**^	0.34^**^	0.26^**^	1	
6. Hope for the protests	5.05	2.38	0.52^**^	0.51^**^	0.80^**^	0.65^**^	0.28^**^	1

M and SD are used to represent mean and standard deviation, respectively. ^*^ indicates *p* < 0.05 and ^**^ indicates *p* < 0.01.

On the other hand, we also found a significant association between non-normative political action with antagonistic negative emotions toward police repression *r*(317) = 0.53, *p* < 0.01, negative feelings of resignation toward police repression *r*(317) = 0.33, *p* < 0.05 and hope toward protests *r*(317) = 0.52, *p* < 0.01.

Based on these results, we can establish that the variables are independent, which allows us to conduct mediation analyses that investigate the role of emotions as explanatory variables of normative and non-normative political action.

#### 3.2.1. Mediation analysis

##### 3.2.1.1. Model 1. Normative political action

**Figure 2:**
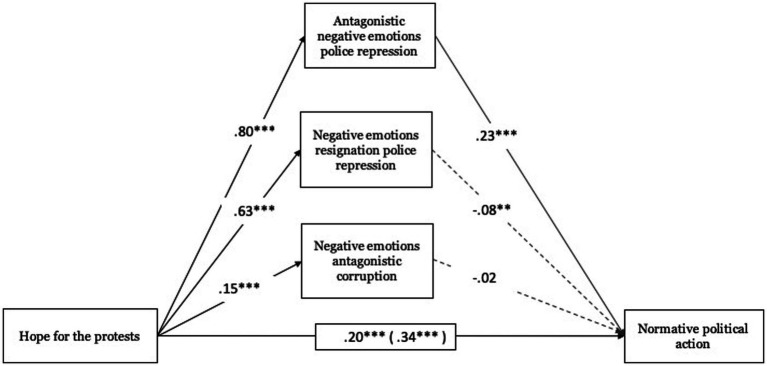
Mediating role of emotions towards police repression and corruption on normative political action. Unstandardized regression coefficients are displayed. Total effect in parentheses. ***p < 0.001, **p < 0.01, *p < 0.05, +*p* < 0.10.

Regarding hope toward protests, these were related to antagonistic negative emotions toward police repression (*B* = 0.80, ET = 0.03, *t* = 23.93, *p* < 0.001, 95% CI [0.730, 860]), with the negative emotions of resignation toward police repression (*B* = 0.63, ET = 0.04, *t* = 15.29, *p* < 0.001, 95% CI [0.550, 0.713]) and with the antagonistic negative emotions toward corruption (*B* = 0.15, ET = 0.03, *t* = 5.08, *p* < 0.001, 95% CI [0.092, 0.208]). With respect to antagonistic negative emotions toward police repression (*B* = 0.23, ET = 0.06, *t* = 3.58, *p* < 0.001, 95% CI [0.103, 0.353]) these were significantly associated with normative political action (see Figure 2).

In relation to the total effect, hope toward protests was related to normative political action (Total effect: *B* = 0.34, ET = 0.03, *t* = 10.83, *p* < 0.001, 95% CI [0.280, 0.405]), an effect that was also significant when including mediating variables (direct effect: *B* = 0.20, ET = 0.05, *t* = 3.79, *p* < 0.001, 95% CI [0.095, 0.300]). In one case, the indirect effect test, based on the Bootstrap procedure, was significant. Hope toward protests through antagonistic negative emotions toward police repression (*B* = 0.18, Boot ET = 0.06, 95% CI [0.070, 0.290]) on normative political action presents a significant indirect effect.

##### 3.2.1.2. Model 2. Non-normative political action

**Figure 3:**
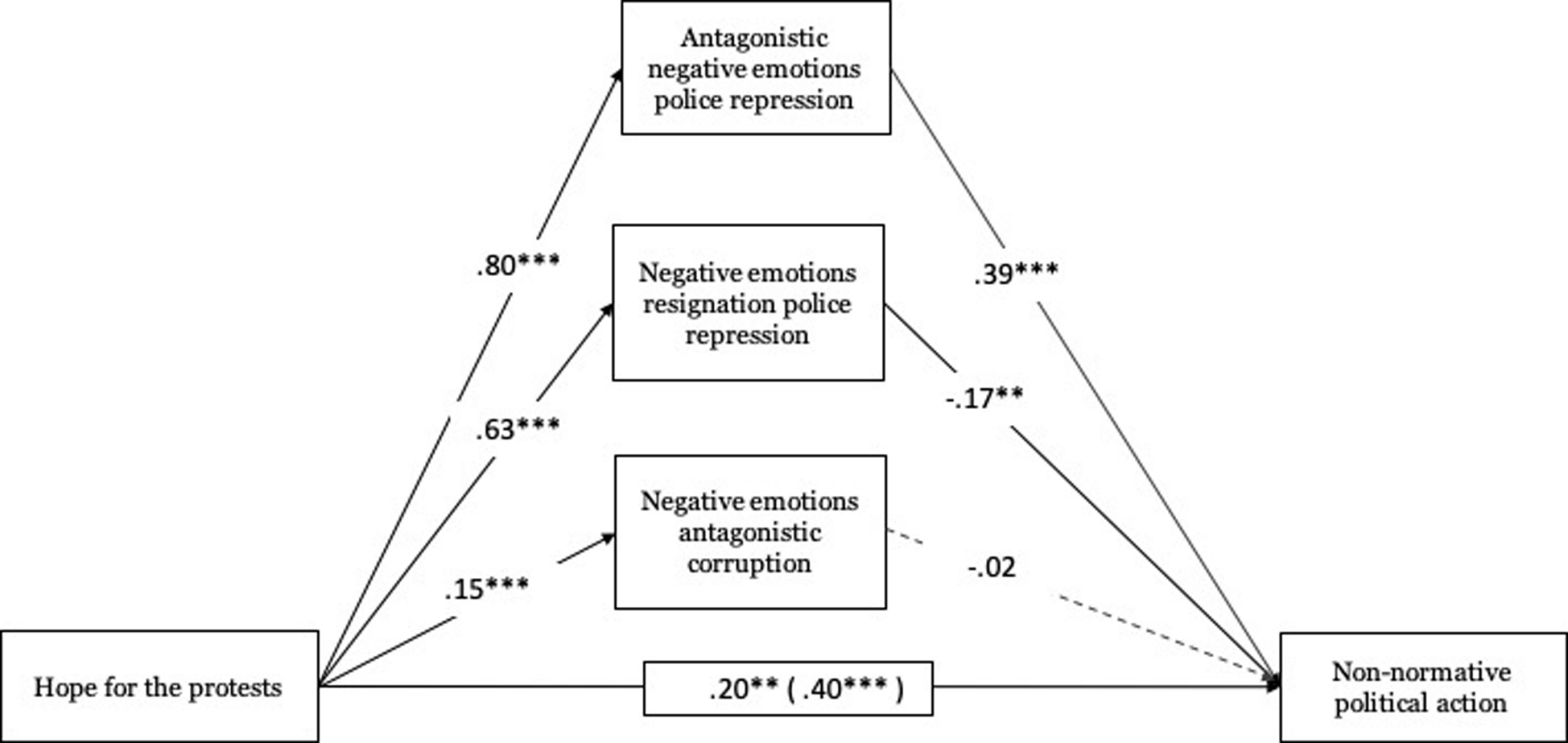
Mediating role of emotions towards police repression and corruption on non-normative political action. Unstandardized regression coefficients are displayed. Total effect in parentheses. ***p < 0.001, **p < 0.01, *p < 0.05, +*p* < 0.10.

Regarding hope toward protests, these were related to antagonistic negative emotions toward police repression (*B* = 0.80, ET = 0.03, *t* = 23.93, *p* < 0.001, 95% CI [0.730, 860]), with negative emotions of resignation toward police repression (*B* = 0.63, ET = 0.04, *t* = 15.29, *p* < 0.001, 95% CI [0.550, 0.713]) and with antagonistic negative emotions toward corruption (*B* = 0.15, ET = 0.03, *t* = 5.08, *p* < 0.001, 95% CI [0.092, 0.208]). Antagonistic negative emotions toward police repression (*B* = 0.39, ET = 0.08, *t* = 5.05, *p* < 0.001, 95% CI [0.235, 0.535]) and negative emotions of resignation toward police repression (*B* = −0.17, ET = 0.06, *t* = −2.77, *p* < 0.01, 95% CI [−0.286, −0.049]) were significantly associated with non-normative political action (see Figure 3).

In relation to the total effect, hope toward protests was related to normative political action (Total effect: *B* = 0.40, ET = 0.04, *t* = 10.41, *p* < 0.001, 95% CI [0.324, 0.474]). This effect was also significant when including mediating variables (direct effect: *B* = 0.20, ET = 0.06, *t* = 3.23, *p* < 0.001, 95% CI [0.079, 0.324]). Indirect effects tests based on the Bootstrap procedure were significant in two cases. Both hope toward protests through antagonistic negative emotions toward police repression (*B* = 0.31, Boot ET = 0.06, 95% CI [0.187, 0.440]) and through negative emotions of resignation toward police repression (*B* = −0.11, Boot ET = 0.04, 95% CI [−0.185, −0.027]) present a significant indirect effect on normative political action.

## 4. Study 3

### 4.1. Method

#### 4.1.1. Participants and procedure

The study participants were 210 Chileans with an average age of 42.88 years (SD = 16.40), of whom 37.7% were female, 61.9% were male and 0.4% identified with another gender. The participants were from the Valparaíso region. Participants were invited to fill in an online questionnaire via the SurveyMonkey@platform. Participants were recruited by a quota sampling. That is, the invitation to fill in the survey was distribute through e-mail and using social networks such as Facebook, Instagram, and Twitter. The data collection started on October 18, 2022, after the Chilean constitutional plebiscite, and was extended until December 6 of the same year.

#### 4.1.2. Measures

***Antagonistic negative emotions towards the Chilean political system***

Participants were asked to indicate on a scale ranging from 1 (*not at all*) to 7 (*a lot*) to what extent they experimented “anger,” “annoyance” and “rage” towards the Chilean political system (*α* = 0.85).

***Negative emotions antagonistic towards the protests***

Participants were asked to indicate on a scale ranging from 1 (*not at all*) to 7 (*a lot*) to what extent they experimented “anger,” “annoyance” and “rage” towards the protests (*α* = 0.92).

***Negative emotions of resignation towards the protests***

We asked participants to report on a scale ranging from 1 (*not at all*) to 7 (*a lot*) to what extent they felt “fear,” “dread,” and “nervousness” towards the protests (*α* = 0.92).

***Positive emotions towards protests***

We asked participants to report on a scale ranging from 1 (*not at all*) to 7 (*a lot*) to what extent they felt hope, joy, pride, sympathy and empathy (*α* = 0.93).

***Negative emotions antagonistic towards the triumph of the rejection in the plebiscite***

Participants were asked to indicate on a scale ranging from 1 (*not at all*) to 7 (*a lot*) to what extent they experimented “anger,” “annoyance” and “rage” towards the triumph of the rejection in the plebiscite (*α* = 0.91).

***Negative emotions of resignation towards the triumph of the rejection in the plebiscite***

We asked participants to report on a scale ranging from 1 (*not at all*) to 7 (*a lot*) to what extent they felt “fear,” “dread,” and “nervousness” towards the triumph of the rejection in the plebiscite (*α* = 0.91).

***Positive emotions towards the triumph of the rejection in the plebiscite***

We asked participants to report on a scale ranging from 1 (*not at all*) to 7 (*a lot*) to what extent they felt joy, pride and sympathy (*α* = 0.91).

***Normative political action***

We captured intentions to participate in non-normative action by asking participants about their general willingness to participate in political activities in Chile. Specifically, they were asked to indicate on a scale from 1 (*not willing at all*) to 7 (*extremely willing*) to what extent they would be willing to “Sign a petition,” “Participate in legal/sanctioned demonstrations (marches),” “Give opinions about politics on social networks (Twitter, Facebook, etc.),” and “Vote in municipal, parliamentary or presidential elections.” The scale was reliable (*α* = 0.78).

***Non-normative political action***

Non-normative collective action intentions were measured with other four items. Participants responded on a scale from 1 (*not willing at all*) to 7 (*extremely willing*) to what extent they would be willing to “Support boycotts,” “Participate in illegal strikes (work stoppages),” “Occupy buildings or factories (seizure),” “Participate in violent actions such as throwing stones, burning or breaking urban furniture, barricades, etc..” The scale showed good internal consistency (*α* = 0.80).

#### 4.1.3. Analytical strategy

We used Pearson’s coefficient for correlation analyses. We also used the Process v3.5 macro of SPSS version 24, with the multiple mediation model that simultaneously estimates multiple indirect effects with standard error and confidence intervals derived from the Bootstrap distribution (Preacher and Hayes, 2004).

### 4.2. Results

#### 4.2.1. Correlation analysis

In relation to the association between the variables analyzed, we found a significant correlation between most variables. As Table 4 shows, normative political action was positively associated with antagonistic negative emotions toward the triumph of rejection in the plebiscite *r*(197) = 0.48, *p* < 0.01, with negative emotions of resignation toward the triumph of rejection in the plebiscite *r*(197) = 0.41, *p* < 0.01, and with positive emotions toward protests *r*(196) = 0.35, *p* < 0.01. And it was negatively associated with positive emotions toward the triumph of rejection in the plebiscite *r*(197) = −0.21, *p* < 0.01.

**Table 4:** Means, standard deviations, and correlations.

Variable	M	SD	1	2	3	4	5	6	7	8	9
1. Normative political action	4.09	1.96	1								
2. Non-normative political action	2.10	1.45	0.53^**^	1							
3. Antagonistic negative emotions towards the Chilean political system	4.97	1.84	0.34^**^	0.30^**^	1						
4. Negative emotions antagonistic towards the triumph of the rejection in the plebiscite	4.36	2.26	0.48^*^	0.46^**^	0.43^**^	1					
5. Negative emotions of resignation towards the triumph of the rejection in the plebiscite	3.52	2.13	0.41^**^	0.40^**^	0.37^**^	0.65^**^	1				
6. Positive emotions towards the triumph of the rejection in the plebiscite	1.85	1.69	−0.21^**^	−0.25^**^	−0.12	−0.47^**^	−0.32^**^	1			
7. Negative emotions antagonistic towards the protests	2.71	1.89	−0.09	−0.13	0.17^*^	−0.10	0.01	0.46^**^	1		
8. Negative emotions of resignation towards the protests	2.59	1.74	−0.13	−0.17^*^	0.17^**^	−0.18^**^	0.06	0.37^**^	0.65^**^	1	
9. Positive emotions towards protests	3.27	1.90	0.35^**^	0.30^**^	0.20^**^	0.31^**^	0.35^**^	−0.22^**^	−0.21^**^	−0.03	1

M and SD are used to represent mean and standard deviation, respectively. ^*^ indicates *p* < 0.05 and ^**^ indicates *p* < 0.01.

We also found a positive association between non-normative political action with antagonistic negative emotions toward the triumph of rejection in the plebiscite *r*(194) = 0.46, *p* < 0.01, with negative emotions of resignation toward the triumph of rejection in the plebiscite *r*(194) = 0.40, *p* < 0.01. And it was negatively associated with positive emotions toward the triumph of rejection in the plebiscite *r*(194) = −0.25, *p* < 0.01, and negative emotions of resignation toward the protests *r*(193) = −0.17, *p* < 0.05.

Based on these results, we can establish that the variables are independent of each other, which allows for mediation analysis. This analysis lets us investigate the role of emotions as explanatory and mediating variables of the disposition to political action (normative and non-normative).

#### 4.2.2. Mediation analysis

##### 4.2.2.1. Model 1: normative political action

**Figure 4:**
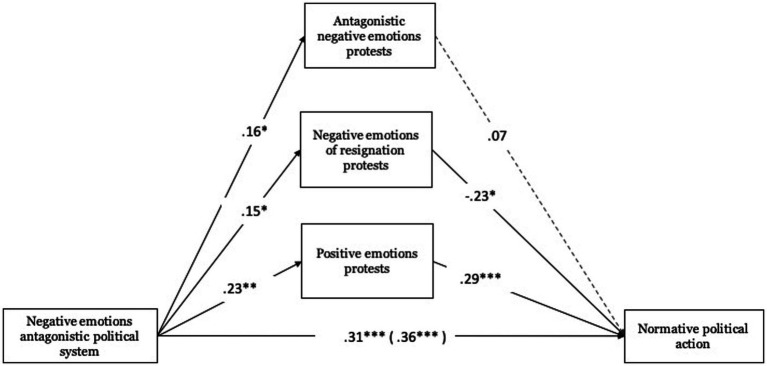
Mediating role of emotions towards protests on normative political action. Unstandardized regression coefficients are displayed. Total effect in parentheses. ***p < 0.001, **p < 0.01, *p < 0.05.

Regarding antagonistic negative emotions toward the political system, these were related to antagonistic negative emotions toward protests (*B* = 0.16, ET = 0.08, *t* = 2.07, *p* < 0.05, 95% CI [0.008, 0.303]), with negative emotions of resignation toward protests (*B* = 0.15, ET = 0.07, *t* = 2.11, *p* < 0.05, 95% CI [0.010, 0.284]), and with positive emotions toward protests (*B* = 0.23, ET = 0.08, *t* = 3.01, *p* < 0.01, 95% CI [0.078, 0.374]). Concerning negative emotions of resignation toward protests (*B* = −0.23, ET = 0.10, *t* = −2.38, *p* < 0.05, 95% CI [−0.414, −0.039]), these were significantly associated with normative political action (see Figure 4).

Concerning the total effect, antagonistic negative emotions toward the political system were related to normative political action (Total effect: *B* = 0.36, ET = 0.07, *t* = 4.84, *p* < 0.001, 95% CI [0.212, 0.503]), an effect that was also significant when including the mediating variables (direct effect: *B* = 0.31, ET = 0.07, *t* = 4.30, *p* < 0.001, 95% CI [0.170, 0.458]). The indirect effect test based on the Bootstrap procedure was significant in one case. Antagonistic negative emotions toward the political system mediated by positive emotions toward protests (*B* = 0.07, Boot ET = 0.03, 95% CI [0.019, 0.125]) present a positive effect on normative political action.

##### 4.2.2.2. Model 2: non-normative political action

**Figure 5:**
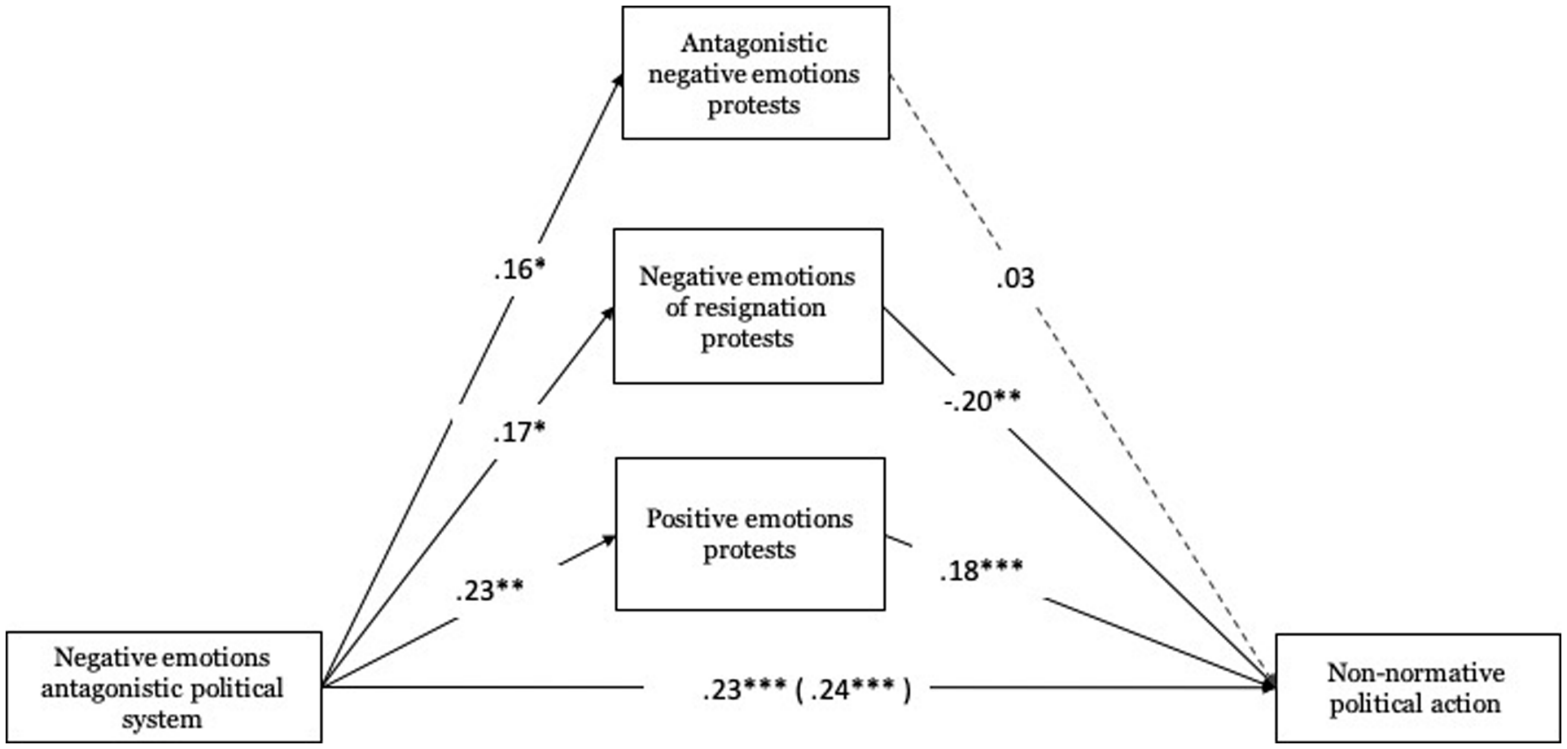
Mediating role of emotions towards protests on non-normative political action. Unstandardized regression coefficients are displayed. Total effect in parentheses. ***p < 0.001, **p < 0.01, *p < 0.05.

Antagonistic negative emotions toward the political system are positively related to antagonistic negative emotions toward protests (*B* = 0.16, ET = 0.08, *t* = 2.05, *p* < 0.05, 95% CI [0.006, 0.306]), with negative emotions of resignation toward protests (*B* = 0.17, ET = 0.07, *t* = 2.45, *p* < 0.05, 95% CI [0.033, 0.306]), and with positive emotions toward protests (*B* = 0.23, ET = 0.08, *t* = 3.02, *p* < 0.01, 95% CI [0.079, 0.379]). Concerning negative emotions of resignation toward protests (*B* = −0.20, ET = 0.05, *t* = −2.63, *p* < 0.01, 95% CI [−0.344, −0.049]) and positive emotions toward protests (*B* = 0.18, ET = 0.05, *t* = 3.02, *p* < 0.01, 95% CI [0.076, 0.288]) these were significantly associated with non-normative political action (see Figure 5).

About the total effect, antagonistic negative emotions toward the political system were positively related to non-normative political action (Total effect: *B* = 0.24, ET = 0.06, *t* = 4.26, *p* < 0.001, 95% CI [0.129, 0.350]). This effect was also significant when including mediating variables (direct effect: *B* = 0.23, ET = 0.06, *t* = 4.06, *p* < 0.001, 95% CI [0.117, 0.338]). The tests of indirect effects, based on the Bootstrap procedure, were significant in two cases. Antagonistic negative emotions toward the political system mediated by negative emotions of resignation toward protests (*B* = −0.03, Boot ET = 0.02, 95% CI [−0.084, −0.003]) exhibit an indirect effect on non-normative political action. As do positive emotions toward protests (*B* = 0.04, Boot ET = 0.02, 95% CI [0.011, 0.082]).

##### 4.2.2.3. Model 3: normative political action

**Figure 6:**
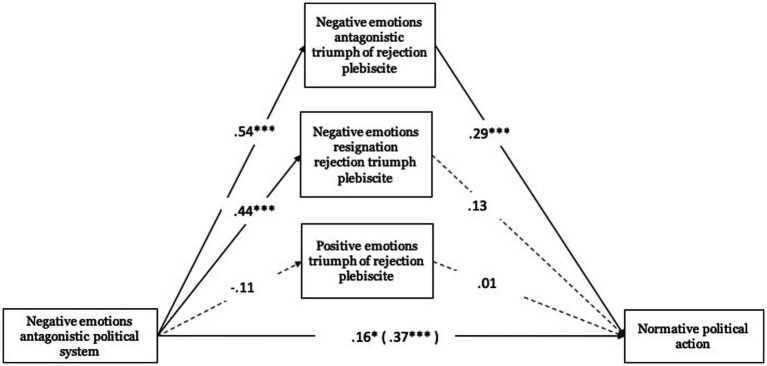
Mediating role of emotions towards the triumph of rejection in the plebiscite on normative political action. Unstandardized regression coefficients are displayed. Total effect in parentheses. ***p < 0.001, **p < 0.01, *p < 0.05.

Regarding antagonistic negative emotions toward the political system, these were related to antagonistic negative emotions toward the triumph of rejection in the plebiscite (*B* = 0.54, ET = 0.08, *t* = 6.60, *p* < 0.001, 95% CI [0.379, 0.701]) and to negative emotions of resignation toward the triumph of rejection in the plebiscite (*B* = 0.44, ET = 0.08, *t* = 5.56, *p* < 0.001, 95% CI [0.284, 0.597]). Concerning antagonistic negative emotions toward the triumph of rejection in the plebiscite (*B* = 0.29, ET = 0.08, *t* = 3.64, *p* < 0.001, 95% CI [0.131, 0.440]), these were significantly associated with normative political action (see Figure 6).

In relation to the total effect, antagonistic negative emotions toward the political system were related to normative political action (Total effect: *B* = 0.37, ET = 0.07, *t* = 5.06, *p* < 0.001, 95% CI [0.225, 0.511]). This effect was also significant when including the mediating variables (direct effect: *B* = 0.16, ET = 0.07, *t* = 2.12, *p* < 0.05, 95% CI [0.011, 0.307]). Also, significant in one case was the bootstrap-based indirect effect test of antagonistic negative emotions toward the political system through antagonistic negative emotions toward the rejectionist triumph in the plebiscite (*B* = 0.15, Boot ET = 0.05, 95% CI [0.063, 0.256]) on normative political action.

##### 4.2.2.4. Model 4: non-normative political action

**Figure 7:**
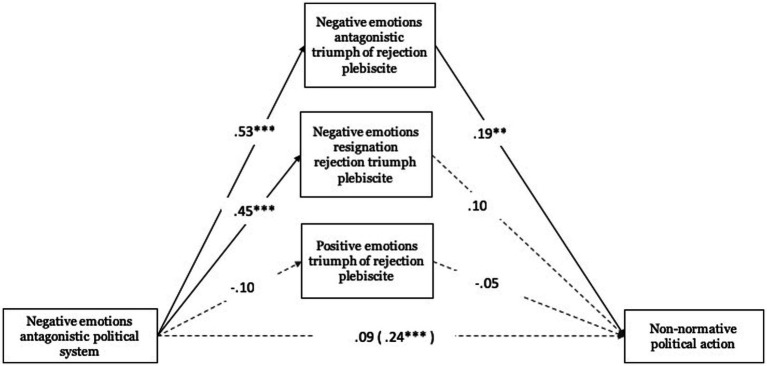
Mediating role of emotions towards the triumph of rejection in the plebiscite on non-normative political action. Unstandardized regression coefficients are displayed. Total effect in parentheses. ***p < 0.001, **p < 0.01, *p < 0.05.

Regarding antagonistic negative emotions toward the political system, these were related to antagonistic negative emotions toward the triumph of rejection in the plebiscite (*B* = 0.53, ET = 0.08, *t* = 6.51, *p* < 0.001, 95% CI [0.372, 0.695]), and to negative emotions of resignation toward the triumph of rejection in the plebiscite (*B* = 0.45, ET = 0.08, *t* = 5.61, *p* < 0.001, 95% CI [0.291, 0.606]). With respect to antagonistic negative emotions toward the triumph of rejection in the plebiscite (*B* = 0.19, ET = 0.06, *t* = 3.13, *p* < 0.01, 95% CI [0.069, 0.305]), these were significantly associated with normative political action (see Figure 7).

Regarding the total effect, antagonistic negative emotions toward the political system were related to normative political action (Total effect: *B* = 0.24, ET = 0.06, *t* = 4.30, *p* < 0.001, 95% CI [0.129, 0.347]). However, this effect was not significant when including mediating variables. The indirect effect test, based on the Bootstrap procedure, was significant in one case. Antagonistic negative emotions toward the political system mediated by antagonistic negative emotions toward the rejectionist triumph in the plebiscite (*B* = 0.10, Boot ET = 0.03, 95% CI [0.038, 0.172]) present an indirect effect on non-normative political action.

## 5. General discussion

The aim of this paper was to analyze the role of negative emotions (antagonistic and resignation) and positive emotions as drivers or inhibitors of people’s dispositions towards political action in Chilean citizens, specifically in their willingness to participate in normative and non-normative actions.

The results show that the disposition to normative political action presents a higher mean than non-normative political action in our three studies. In addition, we observed a progressive decrease in the mean values of both variables in each of the studies. This fact could indicate that Chileans are willing to mobilize politically but in a normative rather than a non-normative way. As we temporarily move away from the social outburst during the post-constitutional plebiscite period, there is less disposition to political action.

Regarding emotions, the results indicate that the highest negative antagonistic emotionality is directed towards police repression (Studies 1 and 2), corruption (Study 2), and the political system (Study 3), all relevant situations in the context of the social outburst and during the subsequent constituent process. There is a negative emotionality of resignation towards police repression; and a low negative emotionality towards protest actions (Studies 1 and 3). Meanwhile, positive emotionality is high towards protests and very low towards police violence.

From these results, it is possible to interpret that the police violence that occurred during the outbreak and in the subsequent mobilizations triggered fear and anger in the population. People also experienced hope for the outbreak and for the protest actions that developed after it. The above confirms what has been described in some studies (Paredes and Valenzuela Fuentes, 2020; Sandoval, 2021) in relation to a coexistence of emotions of different signs during Chilean October, marked by the hope generated by the mobilizations and the anger for the repression and violence.

On the other hand, correlation analyses show a differentiated directionality among the emotions evaluated, according to their sign and the object toward which they were directed. People who experienced anger during the social outburst also experienced anger towards police violence. Similarly, people who experienced fear during the riot also felt fear toward the protests and police violence. On the contrary, those who experienced hope during the social outburst also felt hope towards the protests that occurred after, but not towards police violence, which is consistent with the type of emotional object assessed.

Coincidentally with Jasper’s (2014) studies, these analyses confirm that emotions are not experienced in isolation during complex situations such as protest actions. On the contrary, they are experienced as “moral batteries” in which positive and negative emotions are mixed. Although these first results show the significant relation between different emotions, they do not allow us to determine an explanatory link between emotions and social protest. For this purpose, we explore explanatory models such as multiple linear regressions.

With respect to the hypotheses (H1 and H2), these are fulfilled for both normative and non-normative political action disposition. Antagonistic negative emotions during the social outburst and toward police repression predict disposition to political action – both normative and non-normative – in all models. Relative to positive emotions during the uprising and toward protests, they also predict support for willingness to normative and non-normative political action in all models.

Regression analyses show that the different models that included negative emotions (resignation and antagonism) and positive emotions towards the social outburst, protests, and police repression predict differentially the willingness to participate in normative and non-normative political actions. In all cases, we found a satisfactorily explained variance. However, not all the emotions studied have the same predictive value. In the case of willingness to participate in normative political actions, antagonistic negative emotions, both during the outburst and towards police repression, have a significant effect on the model, with a relevant explanatory power. On the other hand, negative emotions of resignation towards the outbreak, protests, and police repression have no predictive value.

On the other hand, in the case of willingness to participate in non-normative actions, we observed a similar result. Negative antagonistic emotions, both during the outburst and towards police violence, have a relevant effect on increasing the criterion variable. In contrast, negative feelings of resignation toward the protests have no effect. These findings confirm what has been raised by several authors (Jasper, 2014; Reed, 2014; Poma and Gravante, 2018; Ransan-Cooper et al., 2018), who found that there is a relation between emotions and protest actions. In our specific case, we found a greater preponderance of negative emotions but with a relevant role of hope when explaining the disposition to political action.

Regarding hypothesis 3, the mediation analysis results indicate that the higher the level of hope towards the protests, the more participants show more antagonistic negative emotions and resignation towards police repression and more antagonistic negative emotions towards corruption. This fact generates a higher disposition to non-normative political action. In this model, only two variables play a mediating role. On the other hand, the higher the level of hope towards protests, the more participants show more antagonistic negative emotions and resignation towards police repression and more antagonistic negative emotions towards corruption. This fact is associated with a higher disposition towards normative political action. Only one variable plays a mediating role, as would be the case of antagonistic negative emotions towards police repression. Based on these results, hypothesis 3 is partially fulfilled.

Concerning hypothesis 4, mediation analyses results indicate that the higher the level of negative antagonistic feelings towards the political system, the participants show more negative antagonistic emotions and resignation towards the protest and, at the same time, more positive emotions towards the protest. In these models, negative emotions of resignation and positive feelings towards protests play a mediating role with political action, inhibiting or driving it, respectively. Conversely, the higher the level of antagonistic negative emotions towards the political system, the participants show more antagonistic negative emotions and resignation towards the triumph of rejection. This fact generates a higher disposition to normative political action. In this model, antagonistic negative emotions towards the triumph of rejection play a mediating role. Based on these results, the hypothesis is partially fulfilled, given that only some variables play a mediating role.

The results of this study allow us to conclude that emotions directed toward different objects related to the Chilean political process play a relevant role in predicting the disposition to mobilize in a normative and non-normative way. In this sense, antagonistic negative emotionality strongly predicted the relation with the dependent variables. At this point, it is worth noting how negative emotions towards police violence is one of the variables that best explain protest actions, coinciding with theoretical approaches that highlight the importance of this emotion in taking a defiant position against an enemy or authority perceived as threatening (Smith and Lazarus, 1990; Klandermans et al., 2008). As qualitative studies on the outburst show, for many people, especially young people, the impulse that led them to go out and demonstrate arose as a product of the perception of injustice, suffering, and anger, experienced individually or collectively (Rivera-Aguilera et al., 2021).

Finally, positive emotionality (hope) is a dimension that explains the criterion variable (normative and non-normative political action) in all three studies. People associate hope with the inclination to feel inspired, plan for a better future (for oneself and others), and be motivated to change adverse life circumstances (Fredrickson, 2008). This fact is consistent with what has been suggested by authors who describe hope as a sort of “revolutionary accelerator” as it constitutes an outlet for negative emotions such as anger, indignation, and even fear (Reed, 2004). As some qualitative studies describe, during the social outburst, expectations of change and images of better futures made it possible for discontent and grief to turn into experiences of articulation experienced as joy and hope (Zarzuri et al., 2021). During the Chilean October, this circumstance is clearly illustrated in the hopeful air that the feminist movement and the performance of LasTesis provided for the mobilizations of the revolt.

These results are consistent with other studies conducted in Chile on the social outburst. They recognize the relevant role of anger or rage as the emotion that explain the willingness to participate in protests; and the role of hope as the emotion that explains the maintenance of mobilizations over time (Asún et al., 2021). That is, we could propose that the anger that arises in the face of the grievances suffered by the members of the group with which we identify ourselves explains the basic motivation to participate; while the hope that floods us when we participate in a collective experience allows us to understand the processes of maintenance and decline of protest processes. Therefore, we can argue that both emotions are relevant to explain theoretically the willingness to demonstrate during the social outburst.

This study has limitations. First, any research on emotions, especially when using self-report, is limited by the ability of participants to recall and verbally report feelings (Lizarazo Pereira et al., 2022). This research is not exempt from this restriction. However, by conducting different studies in different samples but with similar emotion scales and high reliability, we reduced the effects of this limitation. Secondly, from a sociological point of view, there is a need to extend the application of the scale to territorial realities other than Chile, relating political action to other national profiles. Regarding the sampling process, we can say that, although the samples used in the three studies are non-probabilistic, which implies limitations in extrapolating their data, efforts were made in the sampling process to maintain a proportional balance in the main sociodemographic variables, thereby ensuring minimal bias. Additionally, the aim was to incorporate observation units into the sample that exhibit high diversity in their political choices as well as their social class, which collectively enhances the representativeness and heterogeneity of the units of analysis. Finally, from a methodological point of view, it is necessary to deepen into some dimensions that are not sufficiently differentiated in the used subscales of political action, for example, non-normative actions of a violent nature and their potential differentiation in a subscale within this type of political action (Delfino and Zubieta, 2014; Sandoval et al., 2018).

The social outburst cannot be explained solely based on rational dimensions of collective action. The emotions described in this paper played a central role in its development and maintenance. We could conclude by saying that the social outburst, as an event, generated changes in the way of feeling what had been experienced for decades as a diffuse malaise and transformed it into the concrete emotions that shaped the Chilean October experience.

## Data availability statement

The raw data supporting the conclusions of this article will be made available by the authors, without undue reservation.

## Ethics statement

The studies involving human participants were reviewed and approved by Ethical Committee of the University of Valparaíso. The patients/participants provided their written informed consent to participate in this study.

## Author contributions

FH, JS, XF, J-MG, MB, and PI made a significant contribution to the present study. FH and JS participated in the conception and design. FH, JS, and XF also contributed to the writing of the document and the elaboration of the theoretical framework. J-MG and PI worked on the critical review of the article and wrote sections of it. FH, J-MG and MB contributed to specialized data analyses. All authors contributed to the article and approved the submitted version.

## Funding

This work was supported by the FONDECYT Iniciación 11180664; Centro de Estudios Interdisciplinarios en Cultura Política, Memoria y Derechos Humanos de la Universidad de Valparaíso.

## Conflict of interest

The authors declare that the research was conducted in the absence of any commercial or financial relationships that could be construed as a potential conflict of interest.

## Publisher’s note

All claims expressed in this article are solely those of the authors and do not necessarily represent those of their affiliated organizations, or those of the publisher, the editors and the reviewers. Any product that may be evaluated in this article, or claim that may be made by its manufacturer, is not guaranteed or endorsed by the publisher.

## Footnotes

^1^The Chilean constitutional plebiscite was a referendum held on Sunday, September 4, 2022, to determine whether the citizens agreed with the proposed Political Constitution of the Republic drafted by the Constitutional Convention (which operated between July 4, 2021, and July 4, 2022). The plebiscite was about to approve or reject the new Constitution. Approximately 13 million Chileans voted in the referendum, with the rejection triumphing with 61.8% of votes over 38.13% of the approval.
